# Estrogen Enhances the Expression of the Multidrug Transporter Gene *ABCG2*—Increasing Drug Resistance of Breast Cancer Cells through Estrogen Receptors

**DOI:** 10.3390/ijms18010163

**Published:** 2017-01-14

**Authors:** Fung-Wei Chang, Hueng-Chuen Fan, Jui-Ming Liu, Tai-Ping Fan, Jin Jing, Chia-Ling Yang, Ren-Jun Hsu

**Affiliations:** 1 Department of Obstetrics & Gynecology, Tri-Service General Hospital, National Defense Medical Center, Taipei 114, Taiwan; doc30666@gmail.com; 2 Department of Pediatrics, Tungs’ Taichung MetroHarbor Hospital, Taichung 435, Taiwan; fanhuengchuen@yahoo.com.tw; 3 Division of Urology, Department of Surgery, Taoyuan General Hospital, Ministry of Health and Welfare, Taoyuan 333, Taiwan; mento1218@gmail.com; 4 Department of Pharmacology, University of Cambridge, Cambridge CB23 8AQ, UK; tpf1000@cam.ac.uk (T.-P.F.); jinjing1207@hotmail.com (J.J.); cly2375@gmail.com (C.-L.Y.); 5 Graduate Institute of Life Sciences, National Defense Medical Center, Taipei 114, Taiwan; 6 Department of Pathology and Graduate Institute of Pathology and Parasitology, Tri-Service General Hospital, National Defense Medical Center, Taipei 114, Taiwan; 7 Graduate Institute of Life Sciences, National Defense Medical Center, Taipei 114, Taiwan; 8 Biobank Management Center of Tri-Service General Hospital, National Defense Medical Center, Taipei 114, Taiwan

**Keywords:** breast cancer, mitoxantrone (MX), ATP-binding cassette sub-family G member 2 (ABCG2), estrogen, estrogen receptor α (ERα)

## Abstract

Background: Multidrug resistance is a major obstacle in the successful therapy of breast cancer. Studies have proved that this kind of drug resistance happens in both human cancers and cultured cancer cell lines. Understanding the molecular mechanisms of drug resistance is important for the reasonable design and use of new treatment strategies to effectively confront cancers. Results: In our study, ATP-binding cassette sub-family G member 2 (ABCG2), adenosine triphosphate (ATP) synthase and cytochrome c oxidase subunit VIc (COX6C) were over-expressed more in the MCF-7/MX cell line than in the normal MCF7 cell line. Therefore, we believe that these three genes increase the tolerance of MCF7 to mitoxantrone (MX). The data showed that the high expression of COX6C made MCF-7/MX have more stable on mitochondrial membrane potential (MMP) and reactive oxygen species (ROS) expression than normal MCF7 cells under hypoxic conditions. The accumulation of MX was greater in the ATP-depleted treatment MCF7/MX cells than in normal MCF7/MX cells. Furthermore, E2 increased the tolerance of MCF7 cells to MX through inducing the expression of ABCG2. However, E2 could not increase the expression of ABCG2 after the inhibition of estrogen receptor α (ERα) in MCF7 cells. According to the above data, under the E2 treatment, MDA-MB231, which lacks ER, had a higher sensitivity to MX than MCF7 cells. Conclusions: E2 induced the expression of ABCG2 through ERα and the over-expressed ABCG2 made MCF7 more tolerant to MX. Moreover, the over-expressed ATP synthase and COX6c affected mitochondrial genes and function causing the over-expressed ABCG2 cells pumped out MX in a concentration gradient from the cell matrix. Finally lead to chemoresistance.

## 1. Introduction

Breast cancer is the most common cancer in females. The annual incidence rate is about 1,150,000 new cases and 370,000 deaths worldwide every year [1]. Approximately one third of women with breast cancer develop metastases and ultimately die from the disease. The most common metastatic development sites include the liver, lung, lymph nodes and bone.

Despite the cancer being responsive to a wide variety of single and combination chemotherapy regimens, the response rate to subsequent chemotherapy regimens is significantly decreased due to the development of chemoresistance to multidrug resistance (MDR) [2], which is caused by the multidrug transporter (MDT). Advances in elucidating the molecular basis of MDR show that these transporters recognize diverse chemical substrates and reduce the intracellular concentrations of these substrates, leading to treatment failure [3]. Therefore, MDR is a major obstacle in the successful therapy of breast cancer. Although MDR expression is currently evaluated as a prognostic factor in untreated and relapsing tumors [4,5,6], it is unknown how MDR develops in breast cancer.

Mitoxantrone (MX), a synthetic anthracenedione, has attained clinical approval and has been routinely used alone or in combination for the treatment of breast cancers [7,8]. The anti-neoplastic activity of MX is believed to be related to its ability to bind DNA and inhibit DNA topoisomerase II, an essential enzyme in DNA synthesis and meiotic division which is highly expressed in cancer cells [9]. It is known to induce crosslinks and double-strand breaks in DNA, resulting in a breakdown of the transcription and replication, and the generation of reactive oxygen species (ROS) [10,11,12]. Although MX confers a significant benefit in progression-free and overall survival compared with other chemotherapeutic agents [13], only up to a 20% [14] response rate was found in metastatic breast cancer with MX treatment, suggesting that chemoresistance may be present in cancer cells. Hence, it is critical to understand how such chemoresistance is generated.

ABCG2, the subfamily G of the large human adenosine triphosphate (ATP)-binding cassette (ABC) transporter superfamily, known as the breast cancer resistance protein (BCRP) [15], or the ABC transporter in placenta (ABCP) [16], is considered as the main contributing factor to drug resistance in MCF-7/MX cells [17,18]. ABCG2 is a half transporter and it can dimerize with itself (homodimerize) or with other members of the ABCG subfamily (heterodimerize) to function as an efflux transporter [19,20]. ABCG2 is endogenously expressed in various tissues with a barrier function, including the placenta, prostate, small intestine, brain, colon, liver, mammary glands, and kidney, to protect tissues against toxic compounds, drugs and drug metabolites [21,22]. Although limited clinical data are available as of yet, the widespread expression of ABCG2 and resistance to several structurally irrelevant chemicals suggest ABCG2 may possibly involve innate or acquired resistance of tumors to anti-neoplastic drugs, clinically [23].

The acquired resistance of tumors always arises following therapy and the clinical efficacy of several anticancer drugs has been compromised by the appearance of drug-resistant MDR cells. Therefore, understanding how MDR develops can result in more specific and effective therapeutic approaches for patients. Although ABCG2 is believed to be an efflux transporter for pumping out intracellular medications and toxins, cells transfected with ABCG2 were not resistant to MX [24], suggesting that resistance to MX is complicated and multifactorial. Many clinical experiences have shown that the breast cancer patients whom had ER-positive were easier to treat, as they have a tolerance to MX; therefore, we believe estrogen has a great relationship with MDR in the breast cancer cell line. In our research, we used a c-DNA array to analyze the sensitivity and tolerance of MCF7 (MCF7/MX) to MX. The results show that the expression of ABCG2, COX6C, and ATP synthase increased in MCF7/MX cells. ABCG2 increased after the stimulation of estrogen and the tolerance to MX also changed through the different estrogen concentrations. Thus, the data indicated that estrogen increased the tolerance of breast cancer cells to MX through ER.

## 2. Results

### 2.1. Characterization of MCF-7 and MCF-7/MX Cells

**Figure 1:**
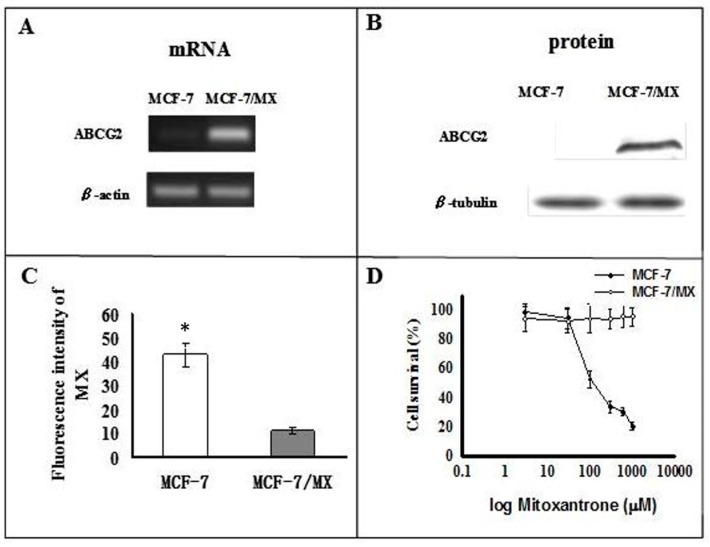
MX resistance and over-expression of ABCG2 in MCF-7/MX cells. Level of (**A**) mRNA and (**B**) protein of ABCG2 in MCF-7 and MCF-7/MX cell lines; (**C**) Retention of MX in MCF-7 cells and MCF-7/MX cells. Fluorescence was measured using the FacsCalibur flow cytometer (Becton–Dickinson, Mountain View, CA, USA) (*n* = 4). (* *p* < 0.001); (**D**) Chemosensitivity of MCF-7 and MCF-7/MX cells to mitoxantrone. Cell viability was determined by MTT assay when cancer cells were seeded onto 96-well plates and treated in triplicate with serially diluted mitoxantrone at 3, 30, 100, 300, and 1000 µM (*n* = 4).

mRNA and protein of ABCG2 were over-expressed in the MCF-7/MX cells compared to the MCF-7 cells (Figure 1A,B). Functionally, flow cytometry showed that intracellular accumulation of MX was much lower in MCF-7/MX cells compared to MCF-7 cells (Figure 1C). As expected, the MTT assay showed that the survival rates of MCF-7/MX cells were much higher than MCF-7 cells after treatment with MX (Figure 1D). These findings are compatible with the idea that MCF-7/MX acquired resistance to MX by actively extruding MX through over-expressed ABCG2 [25].

### 2.2. Microarray Analysis

**Figure 2:**
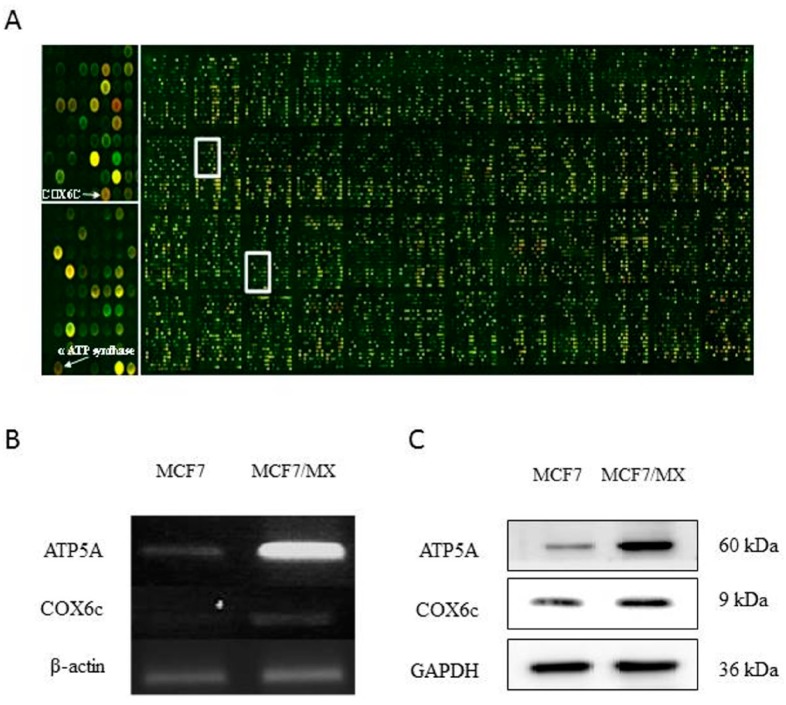
The hybridization on the microarray chip. (**A**) Identification of genes potentially involved in the multidrug resistance (MDR) phenotype. RNA from MCF-7 cells and MCF-7/MX cells was reverse transcribed and labeled with Cy3-dCTP (MCF-7; **green**) and Cy 5-dUTP (MCF-7/MX; **red**). Genes up-regulated in MCF-7/MX appear red and those that are down-regulated appear green. A portion of the cDNA microarray is magnified on the right to show the element representing the ATP synthetase α subunit (α ATP synthase) and *COX6C* genes, which are over-expressed only in the multidrug-resistant MCF-7/MX cells; (**B**) mRNA level of ATP synthetase α subunit (α ATP synthase) and cytochrome c oxidase subunit VIc (COX6C) in MCF-7 and MCF-7/MX cell lines; (**C**) Protein level of ATP synthetase α subunit (α ATP synthase) and cytochrome c oxidase subunit VIc (COX6C) in MCF-7 and MCF-7/MX cell lines.

As angiogenesis correlates closely with tumor growth, metastasis, chemoresistance, and poor prognosis, we used a cDNA microarray designed for angiogenesis to identify changes in gene expression that contribute to the MDR phenotype. The MX-selected MCF-7/MX cells were compared with their MX-sensitive parent MCF-7 cells. These cells were used for microarray hybridization to identify differentially expressed genes. A number of unique human genes (15,164) were examined for differential expression using two-color fluorescence hybridization to the glass cDNA microarray (Figure 2A). The same experiments were performed three times to reduce random variation. We identified 19 up-regulated genes, which, as a result of MX selection, were ranked in descending order of the average of the fold change and these genes were assorted into functional groups based on the information retrieved from the National Center for Biotechnology Information/Entrez/Online Mendelian Inheritance in Man database search (Table 1).

### 2.3. Expression of Mitochondrial-Related Genes

A subset of differentially expressed genes was of particular interest because of their roles in angiogenesis signaling. As 40% of the breast cancers were found to contain hypoxic tissue areas [26] and a clinical study showed that intralesional hypoxia is correlated with higher grade, greater proliferation index, larger tumor size, and resistance to therapeutic agents in patients with breast cancers [25], defects in mitochondrial signaling may provide a clue to the development of chemoresistance. Therefore, ATP synthase and cytochrome c oxidase subunit VIc (COX6C) were selected for further study, and their expression was validated by RT-PCR. It is confirmed that MCF-7/MX cells over-expressed ATPase and COX6C as compared to the parental MCF-7 cells (Figure 2B,C).

### 2.4. Assessment of Mitochondrial Function by Hypoxia Treatment

**Figure 3:**
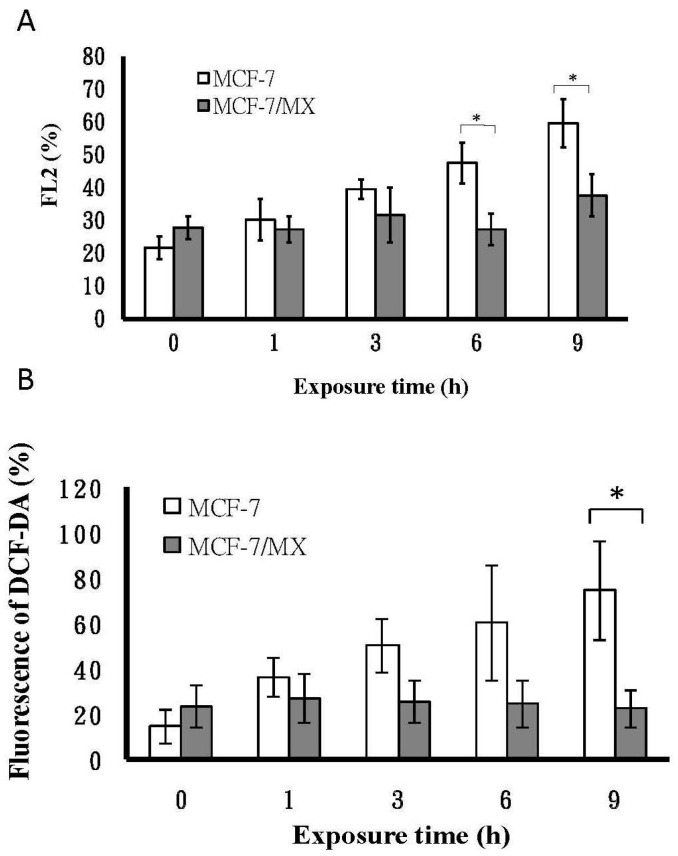
Mitochondrial function of MCF-7 and MCF-7/MX under hypoxia. (**A**) Mitochondrial membrane potential was determined by reduction of JC-1 fluorescence in MCF-7 and MCF-7/MX cells under hypoxia (at 1% oxygen). Data are given as percentage; (**B**) ROS generation in MCF-7 and MCF/MX cells under hypoxia. MCF-7 cells and MCF-7/MX cells were cultured under 1% oxygen tension in a multi-gas incubator (on day 2 with hypoxic periods of 1, 3, 6, and 9 h). An oxygen electrode was used to confirm and calibrate hypoxia treatment. After hypoxia treatment, the production of ROS was measured by using the oxidation-sensitive fluorescent dye DCF-DA. DCF fluorescence intensity was determined on the basis of mean fluorescence of 10,000 cells with FACScan. Values represent mean ± SE of *n* = 3 independent experiments (*n* = 3). * *p* < 0.05 (MCF-7 vs*.* MCF-7/MX).

Since mitochondria are crucial oxygen sensors, we next examined whether mitochondrial dysfunction was induced by hypoxia, and the mitochondrial membrane potential (MMP) was measured before and after hypoxia because MMP is useful for evaluating mitochondrial function [27]. In this study, the time-course of hypoxia (1% oxygen) showed that the mean percent changes in MMP for MCF-7/MX cells remained constant at each time point (mean: 29.70 ± 6.34). However, the values of MCF-7 cells increased significantly from 21.6 ± 3.51, 30.23 ± 6.37, 39.57 ± 2.89, 47.57 ± 6.17, and 59.67 ± 7.37, respectively, suggesting that MCF-7/MX cells with stepwise MX treatment up-regulating COX6c and/or CPS led to a better tolerance of hypoxia than MCF-7 cells (Figure 3A).

### 2.5. The Production of ROS under Hypoxia

Mitochondrial dysfunction is reported to be involved in the modulation of chemoresistance. Importantly, mitochondrial dysfunction was found to induce ROS overproduction [28,29]. We then asked whether the production of ROS was different between cells with and without mitochondrial dysfunction. To establish a more complete picture, we carried out a time-course experiment on the effect of hypoxia on the production of ROS in MCF-7 and MCF-7/MX cells to characterize the differences between these two cell lines. Our data showed that the production of ROS in MCF-7 cells was increased, with a peak occurring after 9 h of hypoxia (Figure 3B). In contrast, there was no obvious change of ROS production in MCF-7/MX cells.

### 2.6. ATP Depletion Increased the Sensitivity of MCF-7/MX Cells to MX

**Figure 4:**
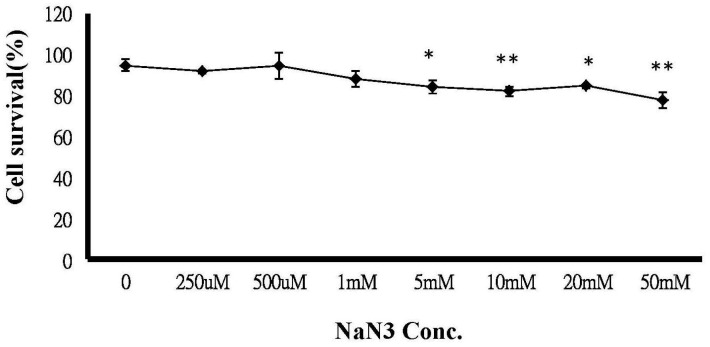
Chemosensitivity of MCF-7/MX cells to MX is significantly affected by NaN3. MCF-7/MX cells were seeded onto 96-well plates. Following 24 h incubation, cells were treated with 4 h ATP depletion with varying concentrations, including 0, 250 and 500 μM 1, 5, 10, 20 and 50 mM sodium azide. Cells were then treated in triplicate with 300 µM. MX cell viability was determined by MTT assay (*n* = 3). The values represent the mean ± standard deviation obtained from triplicate experiments. * *p* < 0.05, ** *p* < 0.005.

**Figure 5:**
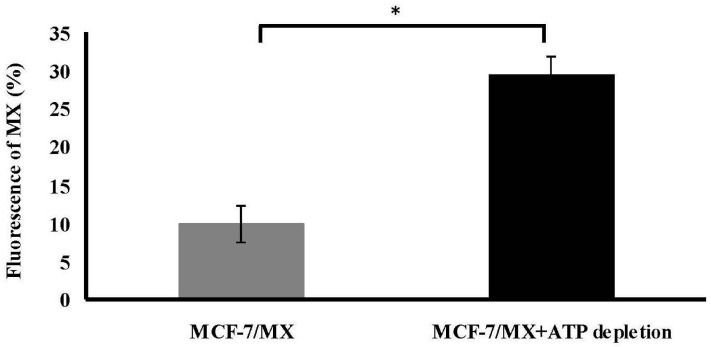
Treatment of depleted ATP efficiently reduced efflux of MX in MCF-7/MX cells. After ATP being depleted 4 h, including incubation in glucose-free and serum-free medium containing 5 mm sodium azide, MCF-7/MX cells were treated with 10 µL MX for 30 min in complete medium. Fluorescence was measured using the FacsCalibur flow cytometer (Becton–Dickinson). Efflux of MX is significantly decreased in MCF-7/MX cells with 4 h ATP depletion (bar in black). The values represent the mean ± standard deviation obtained from triplicate experiments. * *p* < 0.05.

An MTT assay was performed on MCF-7/MX cells following exposure to 300 µM MX, which was the dose showing the distinct MX sensitivity between MCF-7 and MCF-7/MX cells in Figure 1D. The MTT assay showed a dose-related decrease in cell viability in MCF-7/MX cells, which was significantly enhanced by simultaneous treatment with 300 µM MX when the concentration of sodium azide was more than 5 nM (Figure 4). Figure 5 shows the accumulation is decreased in cells treated with ATP depleted. The MX retained in MCF-7/MX cells with and without ATP depletion and 5 nM NaN3 was 29.56% and 9.33%, respectively.

### 2.7. E2 Increased the Drug Resistance to MX by Enhancing the Expression of ABCG2 through ERα in MCF7 Cells

**Figure 6:**
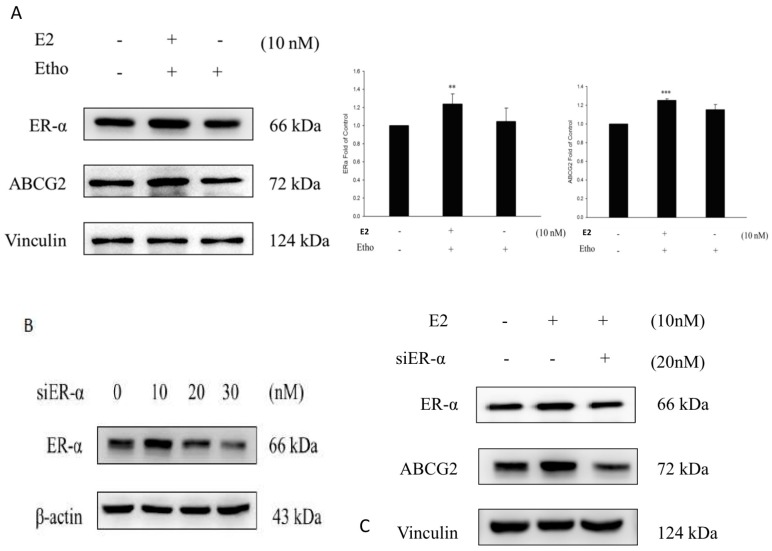
ERα and ABCG2 increased after E2 treatment in the MCF7 cell line. (**A**) After the treatment of E2 in 10 nM, the expression of ERα and ABCG2 increased significantly compared to the control group (ethanol only) (+ represents the addition; - represents not addition); ** *p* < 0.01, *** *p* < 0.001 indicate significant differences compared to the control group; (**B**) The expression of ERα in the MCF7 cell line was inhibited by siERα and the more siERα there was, the lower the ERα expression was; (**C**) siERα inhibited the cell viability of MCF7 cells compared to the control group (ethanol only) after the treatment of 20 nM siERα and 10 nM E2; (+ represents the addition; - represents not addition); The expression of ABCG2 was decreased by siERα through the inhibition of the ERα protein level compared to the control group (ethanol only).

The data showed that E2 increased the expression of ERα and ABCG2 compared to the ethanol treatment group in MCF7 cells (Figure 6A), thus indicated that the expression of ABCG2 was affected by E2. We then used siERα to inhibit the expression of ERα in MCF7 cells, and the data showed that after the inhibition of endogenous ERα by siERα, the expression of ABCG2 did not increase after the E2 treatment (Figure 6C). Therefore, the cell viability of MCF7 cells was reduced because of the decreased expression of ABCG2 (Figure 6B).

**Figure 7:**
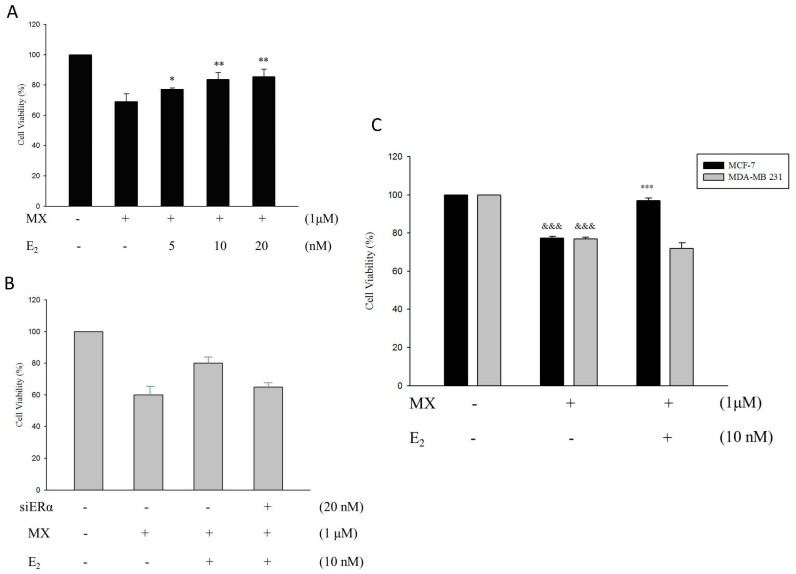
E2 reduces the cell death caused by MX in a dose-dependent manner in normal MCF-7 cell line. (**A**) E2 increased the cell viability in a dose-dependent manner which were co-treated with l μM MX in normal MCF7 cells.* *p* < 0.05, ** *p* < 0.01 indicate significant differences compared to the control group; (**B**) siERα inhibited the cell viability of normal MCF7 cells (l μM MX treatment) compared to the control group (E2 only) (+ represents the addition; - represents not addition); (**C**) E2 was treated with l μM MX in MCF7 cells and MDA-MB231 cells. The cell viability increased 25% in the E2 treatment group compared to the control group (MX only) in normal MCF7 cells while there was no significant difference in MDA-MB231 cells. &&& *p* < 0.001 indicates significant differences compared to the control group and *** *p* < 0.001 indicates significant differences compared to the MX treatment group.

E2 treatment was used on different concentrations of normal MCF7 cells, which were pre-treated with 1 µM MX. We can know, obviously, that the cell viability increased and this means drug resistance to MX increased in normal MCF7 cells (Figure 7A). Additionally, the cell viability decreased significantly in the siERα treatment group compared to normal MCF7 cells without siERα, and the increase of drug resistance to MX stimulated by E2 was inhibited by the decrease of ERα expression (Figure 7B). Then, we co-treated E2 and MX in normal MCF7 cells and MDA-MB231 cells, which lack ERα. The results indicated that E2 can only increase drug resistance to MX in normal MCF7 cells and not in MDA-MB231 cells (Figure 7C). We can make the conclusion that drug resistance to MX was regulated by the protein level of ABCG2, and E2 enhanced the expression of ABCG2 through ERα in MCF7 cells.

## 3. Discussion

In the MCF-7/MX–resistant cell line, the up-regulated ABCG2 requires ATP to support the major mechanism of drug resistance, and mitochondria are mainly responsible for providing cellular energy, and MX cytotoxicity may affect the mitochondrial inner membrane proteins [30] and the mitochondrial respiratory chain [31]. We, hence, investigated mitochondrial-related gene expression and cell functions further.

ATP synthase is a large molecular complex and is embedded in the inner membrane of mitochondria. Its function is for ATP production in oxidative phosphorylation. ATP synthase is reported to be over-expressed in 94.6% of breast cancer samples, and the levels of ATP synthase expression were strongly correlated with large tumor size, poor tumor differentiation, and advanced tumor stage [32]. An early study also found the mRNA levels of αATP synthase were also slightly over-expressed in the adriamycin-resistant breast cancer cell line MCF-7/ADRVP [33], all suggesting a role of αATP synthase in the chemoresistance of breast cancer. Cytochrome c oxidase (COX) is an oligomeric enzymatic complex which is a component of the respiratory chain complex and is involved in the regulation site for oxidative phosphorylation [34]. COX contains 13 different subunits and the function of each subunit is largely unknown. A study using Doxorubicin-resistant leukemia K562 cells found alterations in subunits of COX, suggesting COX might be related to Doxorubicin resistance [35]. Our results validated that the two genes were highly expressed in the resistant cells.

Interestingly, ABCG2 has been detected in the mitochondria, and it plays a significant role in protection against hypoxia. To be validated as a common mechanism of MX resistance in several cell lines, it would be of interest to investigate whether the function of mitochondria is affected in cells with and without over-expression of ABCG2 under hypoxia. MMP is an important parameter in maintaining the stability of the environment of the inner mitochondria and the oxidative phosphorylation pathway. In the present study, we found that alternations of MMP were detected in the MCF-7 cells with higher sensitivity to a chemotherapeutic agent and the resistant strains showed a stable level of MMP when they were challenged with hypoxia. Our findings are in support of the concept that the change of MMP might reflect the extent of restored sensitivity to chemotherapeutic agents in previously resistant cells [36].

The chemoresistance to MX of MCF-7/MX cells was induced by long-term serial passage of the parental wild-type MCF-7 cells in stepwise increasing MX concentrations [14]. Similarly, a glioma resistant to the Temozolomide (TMZ) cell line was generated in the same way. Interestingly, higher ROS production was found in the TMZ-chemoresistant glioma cells under H_2_O_2_ stimulation [37]. However, ROS production under hypoxia showed that the level of ROS increased gradually in MCF-7 cells under hypoxia, and reached a 5.78-fold increase at 9 h when compared to the level at point 0. In contrast, the level of ROS was constant in MCF-7/MX cells under hypoxia. The result might reflect cellular and molecular adaptive changes after long-term, repetitive MX stimulation in MCF-7 cells.

The increased ATP synthase and COX6C in MCF-7/MX cells might be a compensating mechanism to maintain mitochondrial activity so as to decrease the production of ROS and further reducing the apoptotic process, and to act as a survival mechanism to overcome a series of MX treatments. All these arguments were sufficient to prove that ATP synthase and COX6C may be critical factors in the development of resistance to MX in MCF-7 cells.

ABCG2 requires cellular ATP for transporting its substrates and conferring the MDR phenotype [38] to study whether ATP depletion may affect ABCG2 functions and increase chemosensitivity to MX. In fact, modulation of ATP levels has been shown to be therapeutically relevant. Clinical treatments that result in ATP depletion, when used in combination with radiation or chemotherapeutic treatment, appear to lead to enhanced tumor regression in a variety of preclinical studies. However, it is difficult to block drug efflux without altering normal cellular processes.

## 4. Materials and Methods

### 4.1. Reagents

RPMI 1640 medium, fetal bovine serum, penicillin/streptomycin, SuperScript II transcriptase kit, and TaqDNA polymerase were purchased from Invitrogen, Paisley, UK. Mitoxanthrone, TRIZOL reagent, anti-β-tubulin monoantibody, horseradish peroxidase-conjugated anti-mouse IgG, and MTT (3-(4,5-dimethylthiazol-2-il)-2,5-diphenyl tetrazolium bromide) were obtained from Sigma, Dorset, UK. The 2′,7′-dichlorodihydrofluorescein diacetate (H_2_DCFDA) was obtained from Cayman Chemical, Ann Arbor, MI, USA. Anti-HIF-1α and anti-BCRP (BXP-21) antibodies were from Abcam, Cambridge, UK. An enhanced chemiluminescence (ECL) kit was purchased from Amersham Pharmacia Biotech, Uppsala, Sweden.

### 4.2. Cell Culture

The BCRP–over-expressing breast cancer cell line MCF-7/MX and its parental line MCF-7 were kindly provided by M. Barrand (University of Cambridge, Cambridge, UK). These cells were cultured as monolayers in complete medium, including RPMI 1640 medium, 10% heat-inactivated fetal bovine serum (FBS; Gibco BRL, Gaithersburg, MD, USA), 100 U/mL penicillin and 100 μg/mL streptomycin. Further, 80 nM MX was included in the complete medium to maintain the chemo-resistance of MCF-7/MX cells. MCF-7/MX cells were cultured in drug-free medium for at least three days before experiments.

### 4.3. Cytotoxicity Assays

An equal number of MCF-7 and MCF-7/MX cells were inoculated into each well of 96-well plates in triplicate in RPMI 1640 supplemented with 10% fetal bovine serum. After cells attached to the well (36 h), cells were stimulated with 100 µL of serially-diluted MX to achieve final concentrations of 0–1000 μM for an additional 4 h. MTT (Sigma Chemical Co., St. Louis, MO, USA) [27] was then added to each well and was incubated at 37 °C for an additional 4 h. The medium was aspirated from plates leaving about 30 µL of medium in each well. Care was taken not to disturb the formazan crystals at the bottom of the wells. One-hundred fifty microliters of dimethyl sulfoxide (DMSO) was added to each well, and the plates were placed on a shaker for 10 min to solubilize the formazan crystals. The plates were then read immediately at 540 nm on a scanning multiwell spectrophotometer (ELISA reader; Biotek Instruments Inc., Burlington, VT, USA). All of the data points represent the mean value of a minimum of six wells. At least four such experiments were replicated for each cell-line.

### 4.4. Flow Cytometric Detection of Functional Drug Efflux and Accumulation

Exponentially growing MCF-7 and MCF-7/MX cells (10^6^) were incubated for 30 min with 10 µL MX in complete medium and then washed with ice-cold PBS to remove MX-containing medium. The cells were resuspended in drug-free fresh medium and incubated for 90 min. After washed three times with ice-cold PBS, the cells were kept on ice before analysis on a FACSCalibur^TM^ flow cytometer (Becton–Dickinson, Mountain View, CA, USA) equipped with a standard argon laser at 488 nm excitation and with a 530 nm band pass (FL1). Data were analyzed with CellQuest software (Becton–Dickinson, Mountain View, CA, USA).

### 4.5. RNA Extraction and Reverse Transcription-Polymerase Chain Reaction (RT-PCR)

Total RNA was extracted from MCF-7 and MCF-7/MX cells by using TRIzol reagent (Invitrogen Ltd., Paisley, UK) and then treated with DNase (Ambion, Inc., Austin, TX, USA) according to the manufacturer’s instructions. RNA was quantified and quality assessed using a NanoDrop ND-1000 (Nano Drop Technologies Inc., Wilmington, DE, USA) and Agilent 2100 bioanalyzer (Agilent Technologies UK, Stockport, UK), respectively. cDNAs were synthesized by using a SuperScript II reverse transcriptase kit based on the manufacture’s protocol and were used to analyze genes expression after PCR amplification. All PCR products were normalized to β-actin. Primers were designed by using the online “Primer 3” software package (http://bioinfo.ut.ee/primer3-0.4.0/primer3/) and synthesized by MWG Biotech (Buckinghamshire, UK). All used PCR primers, annealing temperatures, and PCR product sizes were listed in Table 1. 5 µg of total RNA was used for generating single-stranded cDNA using the SuperScript Preamplification System (Life Technologies, Inc., Rockville, MD, USA). The cDNA was then used for PCR amplification. The primer sets for PCR amplification were as follows: ABCG2 (product size 429 bps) [30] forward 5′-TTATCCGTGGTGTGTCTGGA-3′, reverse 5′-CCTGCTTGGAAGGCTCTATG-3′; ATP synthase α subunit (product size 171 bps) forward 5′-TTGTGGTGTCGGCTACGG-3′, reverse 5′-CGGCGGAGCAACAGAGA-3′; COX VIc (product size 317 bps) [23] forward 5′-GAAGGACGTTGGTGTTGAGG-3′, reverse 5′-CTAGGGAATTCAACCTGAAG-3′. β-actin (product size 644 bps) [39] forward 5′-50-ACGTTATGGATGATGATATCG-3′, reverse 5′-CTTAATGTCACGCACGATTTC-3′. PCR was carried out with 1 µL of cDNA as follows: initial denaturation at 94 °C for 5 min followed by 25–30 cycles of 94 °C for 30 s, 55–59 °C for 30 s, 72 °C for 1 min, followed by a final elongation at 72 °C for 7 min.

### 4.6. Western Blot Analysis

Cells were lysed in whole lysis buffer (1% Nonidet P-40, 150 mM NaCl, 50 mM Tris, pH 7.5–8.0, 0.02% sodium azide, 0.1% sodium dodecyl sulfate (SDS), 0.5% sodium deoxycholate, 100 μg/mL PMSF, 1 μg/mL aprotinin), and protein concentrations were determined using the Bio-Rad protein assay. Equal amounts of cell lysates were subjected to SDS-polyacrylamide gel electrophoresis, and then transferred to a 0.45 μm nitrocellulose membrane (Amersham, Sweden). Membranes were blocked with 5% skimmed milk-PBS/0.1%. Tween 20 for an hour prior to an overnight incubation at 4 °C with primary antibody (anti-ABCG2, 1:500 and β-tubulin, 1:200, all diluted in 5% skimmed milk in PBS/0.1% Tween 20). The membrane was then incubated with HRP-conjugated secondary antibody. Following successive washes, membranes were developed using an enhanced ECL detection system. β-tubulin was used as an internal control. Anti-ATP5A antibody (ab110273), abcam and Anti-Cytochrome C Oxidase subunit VIc antibody (ab110267), a cam.

### 4.7. cDNA Microarray

#### 4.7.1. Preparation of the cDNA Glass Array

cDNA clones (15,164) for microarray analysis were derived from four sources: (1) 4992 clones from a human testis set IMAGE were purchased from UK Human Genome Mapping Project (HGMP) Resource Centre (MRC Geneservice, Babraham Bioincubator, Babraham, Cambridge, UK); (2) 3360 clones of the HuGen set were also purchased from the HGMP; (3) the Angiogenesis/Apoptosis cDNA set (956 clones) were developed in the Department of Pathology, University of Cambridge; and (4) 5856 cDNA clones came from the Mammalian Gene Collection (available online: http://mgc.nci.nih.gov/). Human cDNA were amplified from bacterial lysates using vector-specific primers and the purified PCR products. They were printed onto GAPSII aminosilane slides (Corning, Corning, NY, USA) in 150 mM phosphate at pH 8.5 and 0.01% SDS buffer. Using a MicroSpot 2500 quill pins (BioRobotics, Cambridge, UK) and BioRobotics 610 Micro-Grid II robot. Spot size was from about 120–160 µm diameters and slides were fixed after printing baking on a hot plate at 80 °C. They were then blocked by immersion in BSA (molecular biology grade B2518; Sigma-Aldrich, St. Louis, MO, USA) and the microarray was washed in SSC and SDS at 65 °C. They denatured by immersion in water at 95 °C for 2 min and slides were immersed in isopropanol before drying by centrifugation.

#### 4.7.2. Preparation of Fluorescence-Labeled Targets and cDNA Microarray Hybridisation and Array Analysis

Total RNAs were transcribed to cDNA and subsequently amplified by switching mechanism at the 5′ end of RNA templates (SMART) methods (BD Biosciences Clontech, Oxford, UK) according to the manufacturer’s protocol. Briefly, samples were amplified by use of the following PCR program: 95 °C for 1 min for 14 cycles, 65 °C for, and 68 °C for 6 min. Twenty-two microliters of amplified cDNA were mixed with 20 µL 2.5× Random Primer, incubated at 95 °C for 5 min, and put on ice. Samples were fluorescence-labeled with 2 µg Cy3-dCTP (MCF-7 cells; green) or Cy5-dUTP (MCF-7/MX; red) using Klenow enzyme (Amersham Biosciences, Buckinghamshire, UK). All labeled cDNAs were purified with Autoseq G50 columns (Amersham Biosciences), pooled with 5 mg/mL human Cot-1 DNA (Life Technologies) and 1 mg/mL Poly dA (Amersham) and hybridized to the cDNA microarray at 50 °C for 16 h. The microarray was washed in 2× SSC and 0.1% SDS for 5 min twice at room temperature with gentle agitation, followed by a 5 min wash in 0.1× SSC twice. The fluorescence signal on microarrays was acquired by using a Genepix 4100 microarray scanner (Axon Instruments, Foster City, CA, USA). Genes up-regulated in MCF-7/MX appear red and those that are down-regulated appear green. The scanned images were processed by using GenePix^®^ Pro 6.0 software (Axon Instrument). Gene expression data were normalized in two ways: per chip normalization and per gene normalization using genespring^®^ 6.0 software (Silicon Genetics, Redwood City, CA, USA) with intensity-dependent (Lowess) normalization (percent of the data used for smoothing = 10%) and per chip normalized to the 50th percentile. Dye swap hybridizations were merged with their counterparts, with the average of the two values for a spot taken as the representative value. Data were filtered with the use of “filter by expression”, “self confidence”, and “Benjamini and Hochberg false discovery test”. Significant genes were selected with a cutoff of *p* < 0.05, a fold change >3, and were further considered for relevance to the MDR phenotype.

### 4.8. Mitochondrial Membrane Potential (MMP) Assay

This assay was modified from a previous report [34]. Briefly, 5,5′,6,6′-tetrachloro-1,1′,3,3′-tetraethylbenzimidazolcarbocyanine iodide (JC-1) (MitoProbe™ JC-1 Assay Kit, Catalog number: M34152, Thermo Fisher Scientific Inc., Waltham, MA, USA) is a mitochondrial-specific and lipophilic cationic dye that undergoes potential-dependent accumulation in the mitochondria. At a higher membrane potential, JC-1 monomers converts to J-aggregates that emit red light (590 nm) following excitation with green light (540 nm). It exists as a monomer when the membrane potential (ΔΨ) is lower than 140 mV and emits green light (540 nm) following excitation by blue light (490 nM).

JC-1 has no affect on living cells, including their respiration [39]. A stock solution of JC-1 of 100 µmol/L was prepared in dimethyl sulfoxide (DMSO). MCF-7 and MCF-7/MX cells (1 × 10^5^) were seeded into 75 cm^3^ flasks. On day 3, cells were treated with 1% oxygen tension in a multi-gas incubator for 1, 3, 6, 9, and 12 h. An oxygen electrode was used to confirm and calibrate hypoxia treatment. After hypoxia treatment, cells were loaded with JC-1 (10 µg/mL) in PBS at 37 °C for 20 min in the dark. Fluorescence was measured using a FacsCalibur flow cytometer (Becton–Dickinson, Mountain View, CA, USA). Changes in the ratio between the red (590 nm) (FL2) and green (540 nm) (FL1) fluorescence intensities indicating the changes in the mitochondrial membrane potential. The ratio of red to green fluorescence is dependent only on the membrane potential but not on other factors such as mitochondrial size, density, cell number and shape [26]. Data was analyzed with the CellQuest 5.1 software (Becton–Dickinson).

### 4.9. Intracellular ROS Analysis

MCF-7 and MCF-7/MX cells (1 × 10^5^) were seeded into 75 cm^2^ flasks. On day 3, cells were treated with 1% oxygen tension in a multi-gas incubator for 1, 3, 6, 9 and 12 h. Oxygen electrode was used to confirm and calibrate hypoxia treatment. After hypoxia treatment, cells were loaded with a fluorescent probe dichlorodihydrofluorescein diacetate (DCF-DA, Molecular Probes (Eugene, OR, USA), 10 μM) [33]. Fluorescence was measured using a FacsCalibur flow cytometer (Becton–Dickinson). To exclude debris, samples were gated based on light-scattering properties in the FSC and SSC modes and 10,000 events per sample within this gate were collected. Data was analyzed with CellQuest software (Becton–Dickinson).

### 4.10. ATP Depletion

To study the effects of ATP depletion (Lobo G.P. ATP modulates PTEN subcellular localization in multiple cancer cell lines, Human molecular genetic 2008), cells were incubated with 37 °C for 4 h in serum-free and glucose-free RPMI 1640 medium containing 10% FBS and 1% penicillin-streptomycin, and sodium azide (NaN3), which affects mitochondrial phosphorylation and, subsequently, lower cellular content. The optimal concentration of NaN3 was determined by the significant cytotoxic effects of MX 300 μM on MCF-7/MX cells. The cells were then placed on ice, the medium removed, and the cell monolayers were washed twice in ice-cold glucose-free HBSS and ready to be used. An ApoSENSOR™ ATP Cell Viability Bioluminescence Assay Kit (BioVision, Catalog number: K254-200) was used to analyze the expression of ATP.

### 4.11. Statistical Analysis

Data are expressed as mean ± SD. A one-way analysis of variance (ANOVA) test was used for comparison of means of normally-distributed parameters. A *p* value less than 0.05 was accepted as significant.

## 5. Conclusions

**Figure 8:**
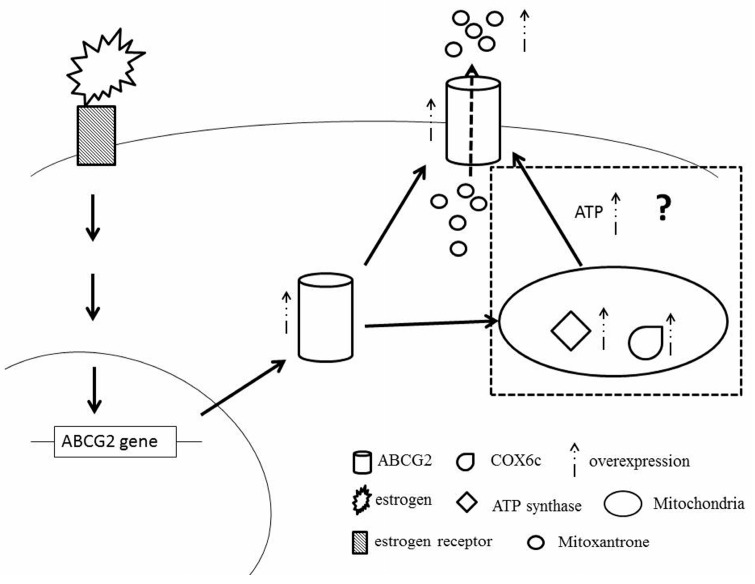
Overview figure. The binding of estrogen and its receptor induced the *ABCG2* gene and the over-expression of ABCG2 modulated MCF7 cells to pump out MX more efficiently. Furthermore, we speculated that the over-expressed ABCG2 imported into mitochondria to stimulate the expression of ATP synthase and COX6c or to interact with over-expressed ATP synthase and COX6c to enhance the tolerance of MCF7 to MX.

In conclusion, MX targets topoisomerase II, generates excessive ROS production, and affects MMP. ABCG2, an energy-dependent drug efflux pump, is considered as the primary, but not the only, contributing factor to drug resistance in MCF-7/MX cells. Changes in mitochondrial functions, including COX6C and ATP synthase, are possibly due to the selection effects of MX on MMP, leading to the over-expression of ABCG2 to pump substrate drugs out of the cells against a concentration gradient. The application of ATP depletion and NaN3 may enhance MCF-7/MX chemosensitivity to MX (Figure 8). The data showed that E2 not only increased the expression of ABCG2 through ER, leading the cell to pump out the MX drug directly, but also induced the highly expressed ATP synthase and COX6c in mitochondria to enhance the tolerance of MCF7 cells to MX. Furthermore, we speculated that the high expression of ATP synthase and COX6c stabilized the mitochondria function and produced a large amount of ATP to help ABCG2 pump the MX out of the cells (Figure 8). In summary, we believe that for the clinical use of MX and other chemotherapy drugs in the treatment of breast cancer, it is important that we consider whether estrogen will increase the tolerance to chemotherapeutic drugs at the same time.

## Figures and Tables

**Table 1:** Genes with elevated expressions in MCF-7/MX cells.

GeneBank/UniGene	Gene Description	Fold Increase	Map
BC008987	Major histocomp atibility complex class II, DR β4	8.3125105	6p21.3
AB009303	Matrix metalloproteinase 16	7.8706665	8q21
AL048744	Clusterin	7.168563	8p21-p12
NM_002456	Mucin 1	5.0657783	1q21
X14787	Thrombospondin 1	5.054978	15q15
BC020968	Chemokine (C-X-C motif) receptor 4	4.8163495	2q21
BC000879	Kynureninase	4.7721705	2q22.3
AI028272	Lymphorid nuclear protein related to AF4	4.69689	2q11.2-q12
X17033	Integrin, α2	3.9517348	5q23-q31
AI0411381	Choline phosphotransferase	3.8714666	12q
NM_003380	Vimentin	3.5217342	10p13
J05633	Integrin, β5	3.2270584	3q21.2
AI025170	Cytochrome c oxidase subunit VIc	3.56	8q22-q23
NM_005147	ATP synthase	3.472383	10p15.1
BC015513	Glutathione S-transferase M4	3.2115557	1p13.3
